# Supplementary material for: A Metabolomics Approach for Early Prediction of Vincristine-Induced Peripheral Neuropathy
Source: Sci Rep. 2020 Jun 15;10:9659. doi: 10.1038/s41598-020-66815-y (PMC7295796; doi:10.1038/s41598-020-66815-y)
Supplement: Supplementary file 1 — Supplementary document. [file 41598_2020_66815_MOESM1_ESM.pdf]

A Metabolomics Approach for Early Prediction of Vincristine-Induced Peripheral Neuropathy–Supplementary document

Parul Verma Jayachandran Devaraj Jodi L. Skiles Tammy Sajdyk Richard H. Ho
Raymond Hutchinson Elizabeth Wells Lang Li Jamie Renbarger Bruce Cooper
Doraiswami Ramkrishna

1 Metabolite selection

In this section, we have provided details on feature selection by recursive feature elimination (RFE). Supplementary Table S1 describes the model accuracy along with sensitivity and specificity for each time point.

After the final feature selection preceded by manual peak integration, models were evaluated using cross validation. Subsequently, models were trained using the complete data and then the threshold for probability was selected. For this selection, various thresholds were tried and specific metrics were evaluated. See Supplementary Fig. S1 for model performance upon changing the probability threshold. Threshold of 0.7, 0.65, and 0.7 was chosen for day 8, day 29, and month 6 models respectively.

After selection of probability threshold, model accuracy was estimated based on the confusion matrices. Confusion matrices for the three time points are shown in Supplementary Table S2.

Supplementary Table S1: Metrics obtained by performing recursive feature elimination on the data sets at the three time points. A. Set of metabolites found that can accurately predict overall neuropathy susceptibility at these time points before manual integration of peaks. B: Set of metabolites found that can accurately predict TNS©-PV intensity at that specific time point. C: Set of metabolites that can accurately predict overall neuropathy susceptibility at the time points after manual integration of peaks. AUROC: Area Under Receiver Operating Characteristics Curve, Sens: Sensitivity, Spec: Specificity, AUROCSD, SensSD, SpecSD are standard deviations for AUROC, sensitivity and specificity. Sensitivity and specificity are calculated by keeping 0.5 probability as the threshold. Note: positive class is overall susceptibility to high neuropathy (HN) for A and C, and TNS©-PV greater than 8 for B.

	Time point	Predictors	AUROC	Sens	Spec	AUROCSD	SensSD	SpecSD
A	Day 8	5	0.968	0.906	0.793	0.048	0.122	0.255
	Day 29	46	0.946	0.936	0.781	0.060	0.090	0.182
	Month 6	42	0.963	0.900	0.918	0.043	0.076	0.152
B	Day 29	2	0.831	0.464	0.865	0.120	0.259	0.092
	Month 6	1955	0.812	0.776	0.617	0.086	0.206	0.204
C	Day 8	6	0.938	0.883	0.677	0.047	0.130	0.261
	Day 29	46	0.861	0.865	0.618	0.122	0.210	0.208
	Month 6	42	0.923	0.844	0.830	0.069	0.119	0.159

Supplementary Table S2: Confusion matrix generated after training the final models with the final selected thresholds for each time point.

Time points	Day 8		Day 29		Month 6	
Reference / Prediction	High	Low	High	Low	High	Low
High	411	32	387	85	362	15
Low	69	128	91	155	118	225

2 Metabolite identification

After the metabolites were finally chosen and models were trained using them, we attempted to identify the metabolites based on their m/z, retention time, MS/MS when available, and adduct information. Supplementary Table S3 shows m/z, retention

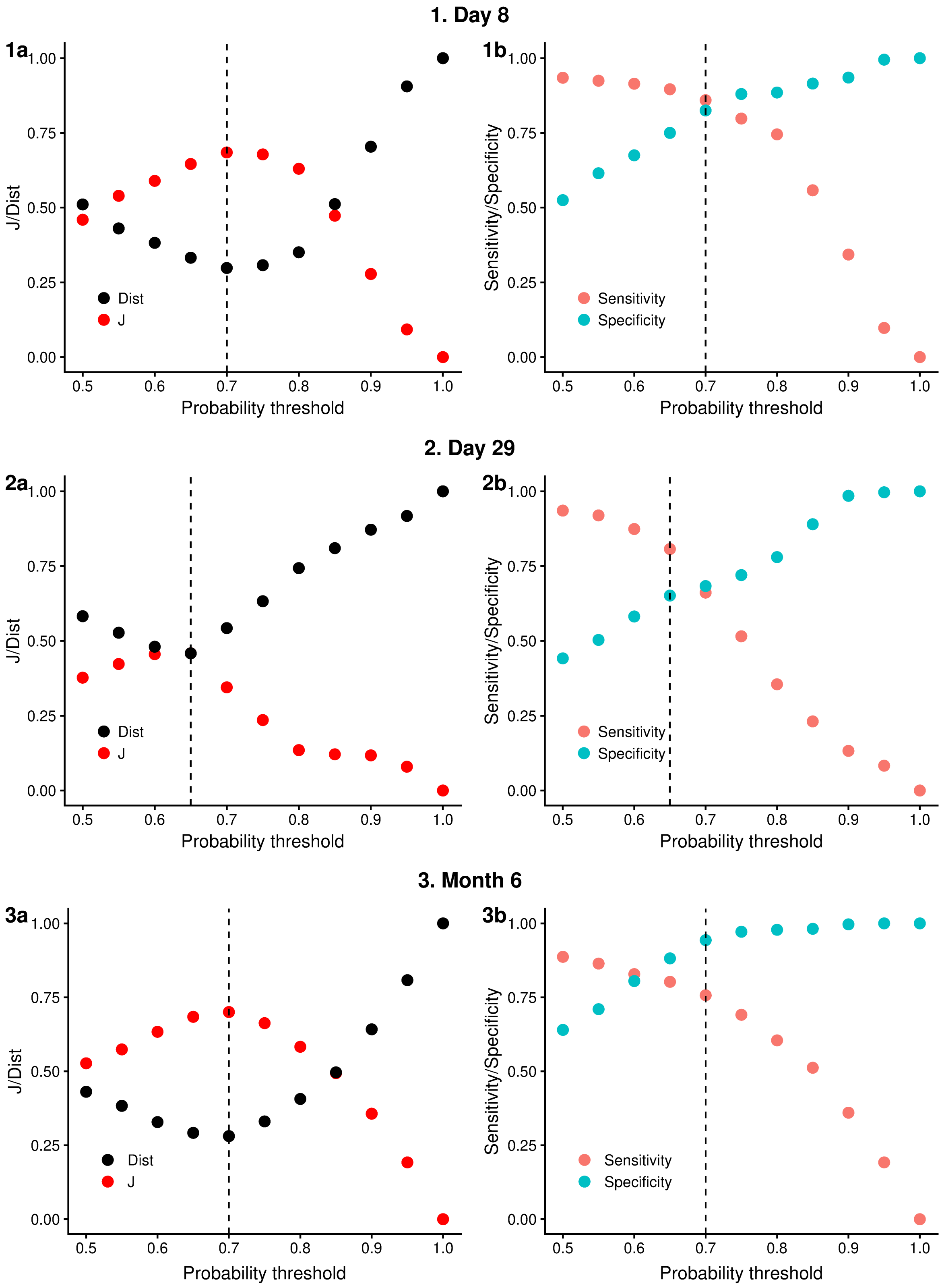

Supplementary Figure S1: The three plots show the metrics evaluated at different probability thresholds. 1a, 2a and 3b show the plot of Youden's J statistic (J) and distance (dist) to best possible cutoff (i.e. sensitivity and specificity equal to 1) at different probability thresholds, at day 8, day 29 and Month 6 data respectively. The probability threshold is for high neuropathy. If the SVM model output is greater than the threshold, the sample is classified as high, and vice versa. 1b, 2b, and 3b show how the sensitivity and specificity varies as a function of probability threshold. Vertical line corresponds to the chosen threshold, based on minimum dist.

time, and adduct information information for the final 2 chosen metabolites. Supplementary Table S4 shows the aforementioned information along with HMDB ID, when possible, for day 29 metabolites. Supplementary Table S5 shows m/z, retention time, adduct information and HMDB ID for month 6 metabolites.

Supplementary Table S3: Mass, retention time, and adduct information for the final set of Day 8 metabolites. None of them could be identified

Mass (Da)	Retention time (min)	Adducts
674.0002	11.99401	Not specified
272.1622	13.85099	M+Na

Supplementary Table S4: Mass, retention time, and adduct information for the final set of day 29 metabolites. 4 of them could be identified.

Mass (Da)	Retention time (min)	Adducts	HMDB ID
174.1012	0.956001	Not specified	HMDB0003357
378.1285	8.478009	Not specified	
188.0794	0.981001	M+H	
228.1216	0.877999	M+H	
508.2309	19.18899	M+Na	
331.3234	19.89402	M+H	
239.2243	18.43899	Not specified	
666.2225	1.045999	M+Na	HMDB0000757
996.6191	19.91397	Not specified	
271.2508	18.65903	M+H	
347.0629	1.77	Not specified	HMDB0000045
320.1222	7.380997	Not specified	
159.0681	4.028006	Not specified	
427.0289	1.273001	2M+H	HMDB0001341

Supplementary Table S5: Mass, retention time, and adduct information for the final set of month 6 metabolites. 9 of them could be identified.

Mass (Da)	Retention time (min)	Adducts	HMDB ID
147.089	0.8	M+H	
129.0789	0.802	M+Na	HMDB0000716
128.0476	5.724	M+H	
385.2012	20.22098	M+H	
700.5516	20.26798	M+H	HMDB0013464
368.1754	20.22302	M+Na	
188.1267	0.710999	Not specified	HMDB0000670
310.1162	5.135994	M+H	HMDB0001961
301.2614	19.797	Not specified	
523.2933	20.203	M+H	
803.5453	20.15502	Not specified	
493.3173	19.99401	M+Na	HMDB0010383
612.152	3.328002	Not specified	HMDB0003337
496.2249	10.36499	Not specified	
294.1104	19.604	M+H	
428.2253	9.986008	M+Na	
260.1371	6.119996	Not specified	HMDB0011170
177.0788	1.277	Not specified	HMDB0001855
414.2041	19.96998	Not specified	
262.1315	8.763993	Not specified	HMDB0011177
208.1098	19.21399	M+H	

3 Pathway analysis

We used Metaboanalyst to perform pathway analysis for the identified metabolites for day 29 and month 6 data. Supplementary Tables S6 and S7 show the results from metaboanalyst for these two time points respectively. None of the pathways were significant.

Supplementary Table S6: Table generated from Metaboanalyst for day 29 metabolites

Pathway Name	Total	Expected	Hits	Raw p	-log(p)	Holm adjust	FDR	Impact
Purine metabolism	65	0.17	2	9.84E-3	4.62	8.27E-1	8.27E-1	0.09
Arginine biosynthesis	14	0.04	1	3.57E-2	3.33	1	1	0

Supplementary Table S7: Table generated from Metaboanalyst for month 6 metabolites

Pathway Name	Total	Expected	Hits	Raw p	-log(p)	Holm adjust	FDR	Impact
Sphingolipid metabolism	21	0.07	1	6.60E-2	2.72	1	1	0
Glutathione metabolism	28	0.09	1	8.72E-2	2.44	1	1	0.03
Glycerophospholipid metabolism	36	0.12	1	1.11E-1	2.20	1	1	0.02
Lysine degradation	25	0.08	1	7.82E-2	2.55	1	1	0

4 Effect of puberty on metabolite expression

We performed hierarchical clustering and attempted to cluster the metabolite profiles based on age groups at the different time points of the treatment (age < 10 and age $>= 10$ years). The dendograms reveal that the samples do not get clustered based on the age groups. Please see Supplementary Figures S2, S3, and S4 for hierarchical clustering performed for the two age groups at different treatment time points. Age is only one of the factors involved in puberty, and we chose age of 10 years as a threshold based on the average age for gonadarche [1]. We focused on age as that is the only variable which was available to us that can be used as a metric for puberty.

We cannot rule out the role of puberty in metabolite expression and developing neuropathy. To address this better, identification of the structures of all the potential biomarker metabolites would have helped. Then, we would have been able to trace the pathways to which they belonged. We could not identify all of them (mentioned in the main manuscript). Among the ones that were identified, the pathways involved were purine metabolism, arginine biosynthesis, sphingolipid metabolism, glutathione metabolism, glycerophospholipid metabolism, and lysine degradation (please see our results on pathway analysis in the main manuscript for this). None of these are directly associated with puberty; the role of puberty is unclear in this case.

References

[1] Jane Mendle, Adriene M. Beltz, Rona Carter, and Lorah D. Dorn. Understanding puberty and its measurement: Ideas for research in a new generation. *Journal of Research on Adolescence*, 29(1):82–95, 2019.

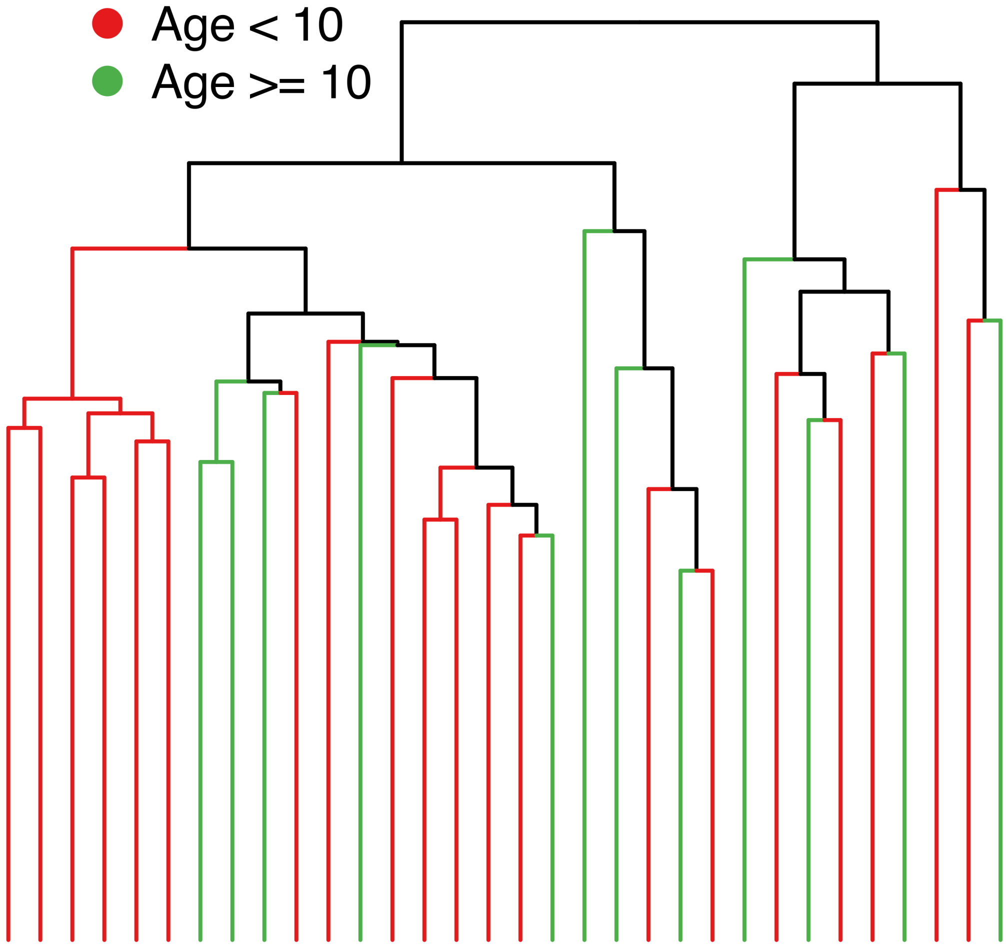

Supplementary Figure S2: Hierarchical clustering dendogram generated for comparing patients of age $<$ 10 and age $>=$ 10 years. Metabolite profiles were compared for day 8 data of these patients.

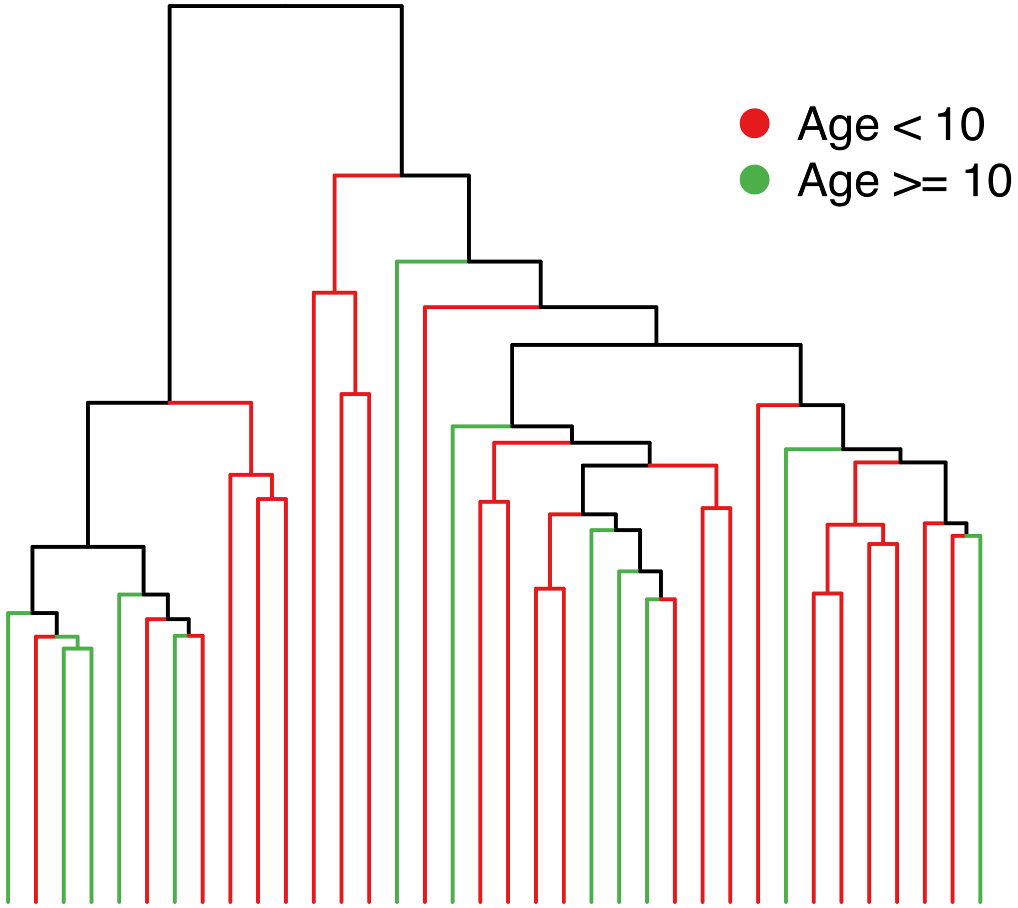

Supplementary Figure S3: Hierarchical clustering dendogram generated for comparing patients of age < 10 and age >= 10 years. Metabolite profiles were compared for day 29 data of these patients.

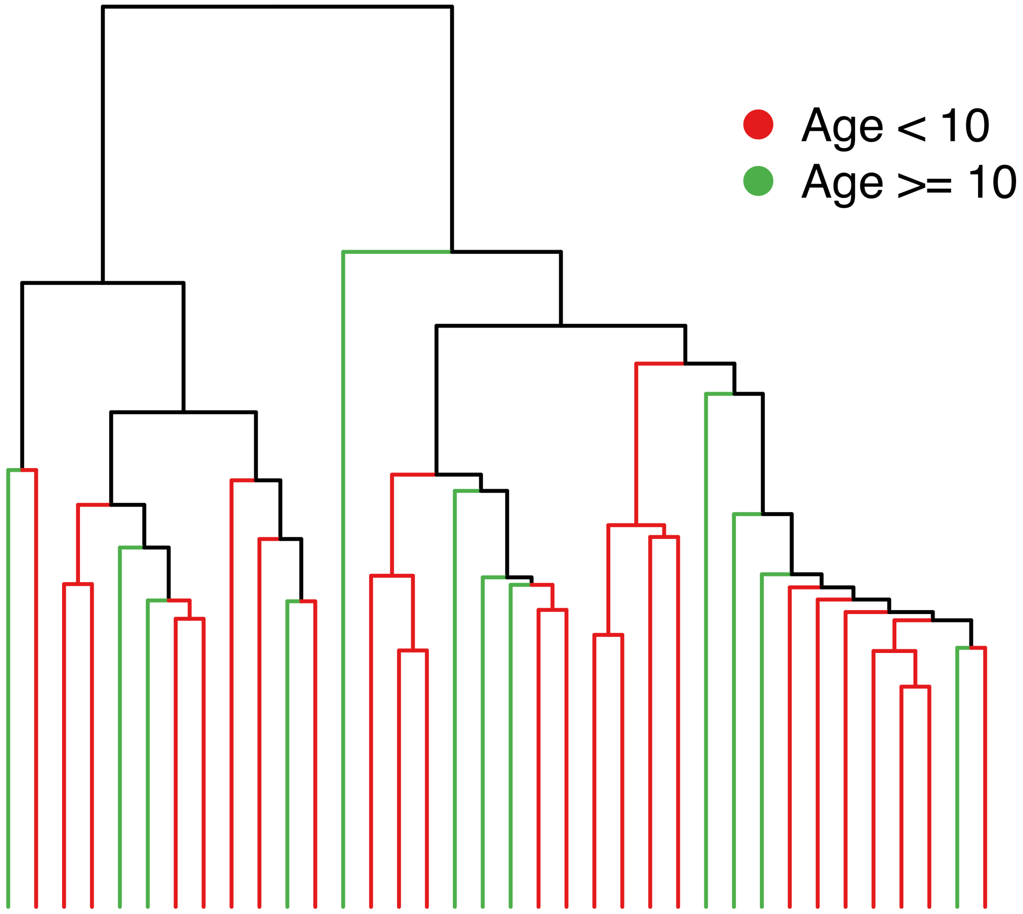

Supplementary Figure S4: Hierarchical clustering dendogram generated for comparing patients of age < 10 and age >= 10 years. Metabolite profiles were compared for month 6 data of these patients.
